# Aliskiren Administration during Early Postnatal Life Sex-Specifically Alleviates Hypertension Programmed by Maternal High Fructose Consumption

**DOI:** 10.3389/fphys.2016.00299

**Published:** 2016-07-12

**Authors:** Chien-Ning Hsu, Kay L. H. Wu, Wei-Chia Lee, Steve Leu, Julie Y. H. Chan, You-Lin Tain

**Affiliations:** ^1^Department of Pharmacy, Kaohsiung Chang Gung Memorial HospitalKaohsiung, Taiwan; ^2^School of Pharmacy, Kaohsiung Medical UniversityKaohsiung, Taiwan; ^3^Institute for Translational Research in Biomedicine, Kaohsiung Chang Gung Memorial Hospital and Chang Gung University College of MedicineKaohsiung, Taiwan; ^4^Department of Urology, Kaohsiung Chang Gung Memorial Hospital and Chang Gung University College of MedicineKaohsiung, Taiwan; ^5^Department of Pediatrics, Kaohsiung Chang Gung Memorial Hospital and Chang Gung University College of MedicineKaohsiung, Taiwan

**Keywords:** aliskiren, fructose, hypertension, renin-angiotensin system, sex differences

## Abstract

**Key points summary**

- Maternal high-fructose (HF) induces programmed hypertension in adult offspring.
- Early aliskiren administration prevents HF-induced hypertension in both sexes of adult offspring.
- HF regulates RAS components in the offspring kidney in a sex-specific manner.
- HF alters renal transcriptome, with female offspring being more sensitive.
- Deprogramming strategy to prevent hypertension might be sex-specific.

**Background:** Maternal high fructose (HF) intake induced renal programming and hypertension in male adult offspring. We examined whether maternal HF intake causes programmed hypertension and whether aliskiren administration confers protection against the process in a sex-specific manner, with a focus on the transcriptome changes in the kidney using next-generation RNA sequencing (NGS) technology and renin-angiotensin system (RAS).

**Methods:** Pregnant Sprague—Dawley rats received regular chow or chow supplemented with 60% fructose throughout pregnancy and lactation. Offspring were assigned to six groups: male control, male HF (MHF), MHF+Aliskiren, female control, female HF (FHF), and FHF+Aliskiren. Oral aliskiren 10 mg/kg/day was administered via gastric gavage between 2 and 4 weeks of age. Rats were sacrificed at 12 weeks of age.

**Results:** Maternal HF intake induced programmed hypertension in 12-week-old offspring of both sexes. HF regulated renal transcriptome and RAS components in the offspring kidney in a sex-specific manner. Aliskiren administration prevented HF-induced programmed hypertension in both sexes of adult offspring. Aliskiren administration increased ACE2 and MAS protein levels in female kidneys exposed to maternal HF intake.

**Conclusion:** Maternal HF induced programmed hypertension in both sexes of adult offspring, which was sex-specifically mitigated by early aliskiren administration. Better understanding of the sex-dependent mechanisms that underlie maternal HF-induced renal programming will help develop a novel sex-specific strategy to prevent programmed hypertension.

## Introduction

Fructose is a natural sugar found in many fruits. Fructose consumption has dramatically increased since the introduction of commercially produced high fructose corn syrup sweeteners. The total per capita use of sweeteners increased by 86% between 1909 and 1997 (Gross et al., 2004). An increase in fructose consumption over the past 2 decades has been linked to a rise in metabolic syndrome comorbidities, such as hypertension (Johnson et al., 2007). Maternal high fructose (HF) intake can lead to hypertension in adult offspring, a phenomenon referred to as developmental programming (Ojeda et al., 2008; Paixão and Alexander, 2013). A shift in the therapeutic approach from the adult to earlier states that precede the development has occurred, a concept called reprogramming (Tain and Joles, 2015b). Although several organs control blood pressure (BP), the developing kidney is particularly vulnerable to the maternal insults. Thus, renal programming has been identified as a driving mechanism of programmed hypertension (Paixão and Alexander, 2013; Tain et al., 2015a; Tain and Joles, 2015b). Previously, we have reported that maternal HF intake induced programmed hypertension in adult male offspring, which can be reprogrammed by early intervention (Tain et al., 2014, 2015d).

Both human and experimental animal evidence shows that hypertension is more common in males compared with females (Sandberg and Ji, 2012) and sex differences also have been observed in some models of programmed hypertension (Sandberg and Ji, 2012; Aiken and Ozanne, 2013). However, it is unclear whether sex differences exist in maternal HF-induced programmed hypertension and reprogramming strategy. Sex differences were reported to be important determinants of the specific pathologies that develop in association with HF intake (Sharma et al., 2015). Sex differences also have been reported in kidney gene expression in F344 rats (Kwekel et al., 2013). Thus, identification of the underlying sex-specific mechanisms could provide novel reprogramming strategy to reach maximal optimization in both sexes. Since nephrogenesis occurs from late gestation to postnatal week 1 in rodents, we therefore employed the whole-genome next-generation RNA sequencing (NGS) to quantify the abundance of RNA transcripts in the kidney of 1-week old offspring of both sexes that had maternal exposure to HF intake, to identify the primary programmed changes.

The renin-angiotensin system (RAS) plays a fundamental role in the regulation of nephrogenesis and blood pressure (BP) control. Treating the young offspring with an ACE inhibitor (ACEI) captopril, angiotensin receptor blocker (ARB) losartan, or renin inhibitor aliskiren between 2 and 4 weeks of age has been reported to offset the effects of developmental programming on BP. These findings support the proposal that targeting the RAS might prevent programmed hypertension (Sherman and Langley-Evans, 1998, 2000; Hsu et al., 2015). The RAS cascade starts with the release of renin in the kidney and sex differences exist in renal RAS expression (Te Riet et al., 2015). The study was therefore designed to address two specific goals: First, to examine if whether early postnatal aliskiren administration can prevent programmed hypertension caused by maternal HF intake in a sex-specific manner; Second, to analyze the renal transcriptome and capture candidate genes and pathways in the offspring kidney exposed to maternal HF intake using NGS approach.

## Materials and methods

### Experimental design

This study was approved by the Institutional Animal Care and Use Committee of the Kaohsiung Chang Gung Memorial Hospital. Experimental animals were treated according to the guidelines of the US National Institutes of Health. Virgin Sprague-Dawley (SD) rats (age, 12–16 weeks) obtained from BioLASCO Taiwan Co., Ltd. (Taipei, Taiwan) were housed in a facility accredited by the Association for Assessment and Accreditation of Laboratory Animal Care International. Rats were exposed to 12-h light/12-h dark photoperiod. Male SD rats were caged with female rats until mating was confirmed by the presence of a vaginal plug. Male rats were removed after mating was confirmed.

Pregnant SD rats received regular chow (*N* = 6) or chow supplemented with 60% fructose throughout pregnancy and lactation (*N* = 6) (Tain et al., 2014). After birth, each litter was left with the mother until weaning; pups were not weighed at birth to prevent maternal rejection. After birth, litters were culled to give equal numbers of males and females for a total of eight pups to standardize the received quantity of milk and maternal pup care. Three male and three female offspring from each group (control and HF) were killed at 1 week of age. Their kidneys were isolated for NGS analysis. The remaining offspring were assigned to 6 groups (*N* = 7/group): male control (MC), male HF (MHF), MHF+Aliskiren, female control (FC), female HF (FHF), and FHF+Aliskiren. Male and female HF offspring in aliskiren groups received oral aliskiren 10 mg/kg/day via gastric gavage (Novartis Pharmaceutical, New York, USA) between 2 and 4 weeks of age. Unlike nephrogenesis in humans, which is completed at 38 gestational weeks it continues into the postnatal weeks 1–2 in rodents. Thus, blockade of the RAS to prevent the programmed hypertension cannot start early until the completion of nephrogenesis in the rodent models (Sherman and Langley-Evans, 1998, 2000; Hsu et al., 2015). The dose of aliskiren used here was based on our previous studies conducted in rats (Tain et al., 2011; Hsu et al., 2015). BP was measured in conscious rats at 3, 4, 8, and 12 weeks of age by using an indirect tail-cuff method (BP-2000; Visitech Systems, Inc., Apex, NC, USA) (Tain et al., 2014). For each rat, five measurements were recorded at each time point. The average of values from three stable measurements was taken. Offspring were euthanized by an i.p. overdose of pentobarbital at 12 week of age. The midline of the abdomen was opened, and the intestines were displaced laterally to allow visualization of the aorta. The aorta was dissected from the adjacent vena cava, connective tissue, and fat. The aorta was cannulated with a 20- to 23-gauge butterfly needle, heparinized blood samples were collected, the vena cava was cut, and PBS was perfused until the kidneys were blanched. Perfused kidneys were harvested, decapsulated, divided into cortex and medulla, flash frozen in liquid nitrogen, and stored at −80°C freezer for further analysis.

### Next-generation sequencing and analysis

Kidney cortex samples (*n* = 3/group) were used for RNA next-generation sequencing (NGS) (Welgene Biotech Co., Ltd., Taipei, Taiwan) using methods described previously (Tain et al., 2015d). Briefly, purified RNA was quantified at OD260nm by using a ND-1000 spectrophotometer (Nanodrop Technology, Wilmington, DE, USA) and qualitated by using a Bioanalyzer 2100 (Agilent Technologies, Inc., Santa Clara, CA, USA) with RNA 6000 labchip kit (Agilent Technologies, Inc.). Library construction was performed using the Solexa platform (Illumina, San Diego, CA, USA). The sequence was directly determined by sequencing-by-synthesis technology using the TruSeq SBS Kit (Illumina). Raw sequences were obtained using the Illumina Pipeline software bcl2fastq v2.0 (Illumina), which was expected to generate 30 million reads per sample. Gene expression was quantified as fragment per kilobase of exon per million mapped fragment (FPKM). The cuffdiff tool (Trapnell et al., 2013) was run to calculate expression changes and associated q values (*P*-values adjusted for false discovery rate) for each gene between MC, MHF, FC, and FHF group. The output files of cuffdiff were further annotated by adding gene functional descriptions and Gene Ontology (GO) classifications. GO analysis for significant genes was performed using Kyoto Encyclopedia of Genes and Genomes (KEGG) and NIH DAVID Bioinformatics Resources 6.7 (NIH, Bethesda, MD, USA) to identify regulated biological themes (Dennis et al., 2003). GO term enrichment and fold enrichment or depletion for gene lists of significantly up- and down-regulated genes in kidney were determined. The reference genome and gene annotations were retrieved from Ensembl database.

### Quantitative real-time polymerase chain reaction

RNA was extracted using TRIzol reagent, treated with DNase I (Ambion, Austin, TX, USA) to remove DNA contamination, and reverse transcribed using random primers (Invitrogen, Carlsbad, CA, USA). Control RT reactions were performed by omitting RT enzyme. RNA concentration and quality were checked by measuring optical density at 260 and 280 nm. Complementary DNA was synthesized using M-MLV Reverse Transcriptase (Invitrogen). Two-step quantitative real-time polymerase chain reaction (PCR) was performed using QuantiTect SYBR Green PCR Kit (Qiagen, Valencia, CA, USA) and iCycler iQ Multi-Color Real-Time PCR Detection System (Bio-Rad, Hercules, CA, USA). Components of the RAS were analyzed including renin (*Ren*), prorenin receptor (*Atp6ap2*), angiotensinogen (*Agt*), angiotensin-converting enzyme (*Ace*), *Ace2*, angiotensin II type 1 (*Agtr1a*) receptor and 2 receptor (*Agtr1b*), and angiotensin (1–7) MAS receptor (*Mas1*). The 18S rRNA gene (*Rn18s*) was used as a reference. Sequences of primers used in this study are provided in Supplementary Table 1. Primer efficiency between 1.8 and 2.2 was acceptable. All the samples were examined in duplicate. Comparative threshold cycle (C_T_) method was used to quantify relative gene expression. ΔC_T_ of each sample was calculated by subtracting its average C_T_ value from the corresponding average value of *Rn18s*. ΔΔC_T_ was calculated by subtracting the average male control ΔC_T_ value from the average experimental ΔC_T_ value. Fold increases in experimental samples relative to the male control sample were calculated using the formula 2^−ΔΔCT^.

### Western blotting

Western blot analysis was performed as we described previously (Tain et al., 2014). We used the following antibodies: for angiotensin converting enzyme 2 (ACE2), rabbit anti-rat ACE2 (1:1000, overnight incubation; Santa Cruz Biotechnology, Santa Cruz, California, USA); for angiotensin II type 2 receptor (AT2R), rabbit anti-rat AT2R (1:250, overnight incubation; Santa Cruz Biotechnology); for angiotensin (1–7) receptor MAS, a rabbit anti-rat MAS antibody (1:1000, overnight incubation; Santa Cruz Biotechnology). Bands of interest were visualized using enhanced chemiluminescence reagents (PerkinElmer, Waltham, MA, USA) and quantified by densitometry (Quantity One Analysis software; Bio-Rad), as integrated optical density (IOD) after subtraction of background. The IOD was factored for Ponceau red staining to correct for any variations in total protein loading. The protein abundance was represented as IOD/PonS.

### Statistical analysis

All data are expressed as mean ± SEM. Parameters were compared using two-way ANOVA followed by a Tukey's *post hoc* test for multiple comparisons. Blood pressures were analyzed by three-way ANOVA, with maternal high-fructose, sex, and aliskiren administration as factors, results and interactions were presented. A *P*-value < 0.05 was considered statistically significant. All analyses were performed using the Statistical Package for the Social Sciences (SPSS) software (Chicago, IL, USA).

## Results

There were no differences in the litter size (control = 11.3 ± 0.7; HF = 11.9 ± 0.9) and ratio of male-to-female pups (control vs. HF = 1.09 vs. 0.94). Pup mortality rate was 0% in each group. As shown in Table 1, HF diet did not significantly affect kidney weight of either sex. There was a significant effect of sex on the body and kidney weights (both *P*_sex_ < 0.01), but not the kidney weight-to-body weight ratio. There was little measureable effect of aliskiren administration on body weight, kidney weight, and the kidney weight-to-body weight ratio in the male or female offspring.

**Table 1:** **Body and kidney weights in the offspring at 12 weeks of age**.

**Groups**		**Controls**	**HF**	**HF+Aliskiren**	***P*****-value**		
					**Sex**	**Aliskiren**	**S × A**
Body weight (g)	Male	458 ± 11	520 ± 18	483 ± 10	< 0.01	0.328	0.616
	Female	262 ± 5	280 ± 10	254 ± 6			
Left kidney weight (g)	Male	2 ± 0.05	1.84 ± 0.08	1.98 ± 0.05	< 0.01	0.743	0.099
	Female	1.12 ± 0.04	1.15 ± 0.04	1.04 ± 0.05			
Left kidney weight/100 g body weight	Male	0.44 ± 0.01	0.35 ± 0.01	0.41 ± 0.01	0.487	0.802	0.306
	Female	0.42 ± 0.01	0.41 ± 0.01	0.41 ± 0.01			

*HF, offspring exposed to maternal high-fructose (HF) intake; HF + Aliskiren, offspring exposed to maternal HF intake plus postnatal aliskiren administration; S × A, interaction of sex × aliskiren; N (pups/litters) = 7/4 per group*.

**Figure 1:**
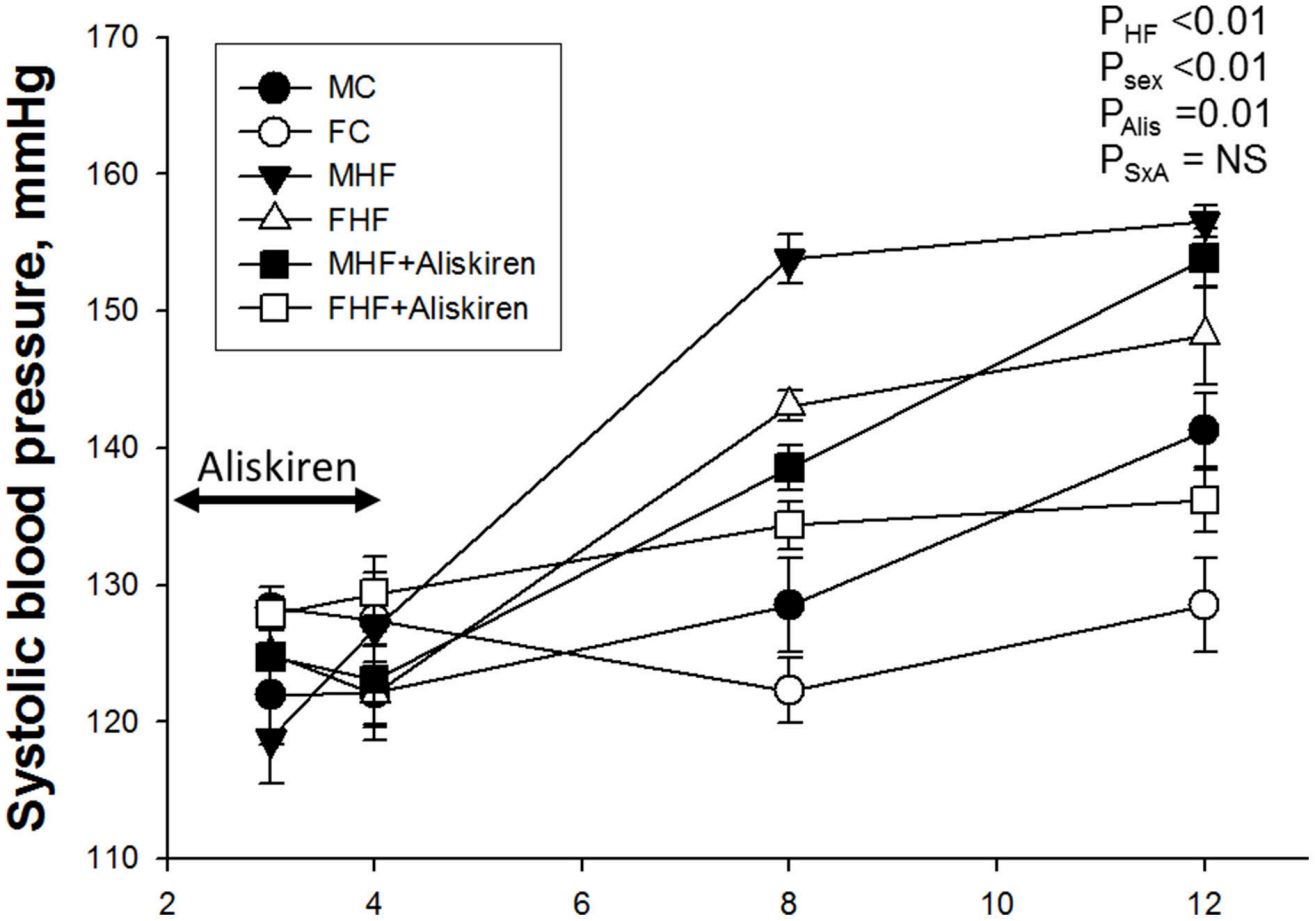
**Effect of maternal high-fructose (HF) and aliskiren on systolic blood pressure in male and female offspring**. *N* (pups/litters) = 7/4 per group; Alis = aliskiren; S × A = interaction of sex × aliskiren.

As shown in Figure 1, the systolic blood pressures (SBP) was lower in the female offspring compared to male offspring (*P*_sex_ < 0.01). The SBP of HF exposure groups were significantly higher than those in the control groups in both sexes from 8 to 12 weeks of age (*P*_HF_ < 0.01). Aliskiren administration reduced SBP in male and female offspring exposed to maternal HF intake at 8 weeks (*P*_Alis_ < 0.01) and 12 weeks of age (*P*_Alis_ = 0.01). However, time-dependent effect of aliskiren administration on SBP is sex-specific, in that the effect after stopping treatment at 4 weeks persists up to 12 weeks in the females, but disappears between 8 and 12 weeks in the males.

**Figure 2:**
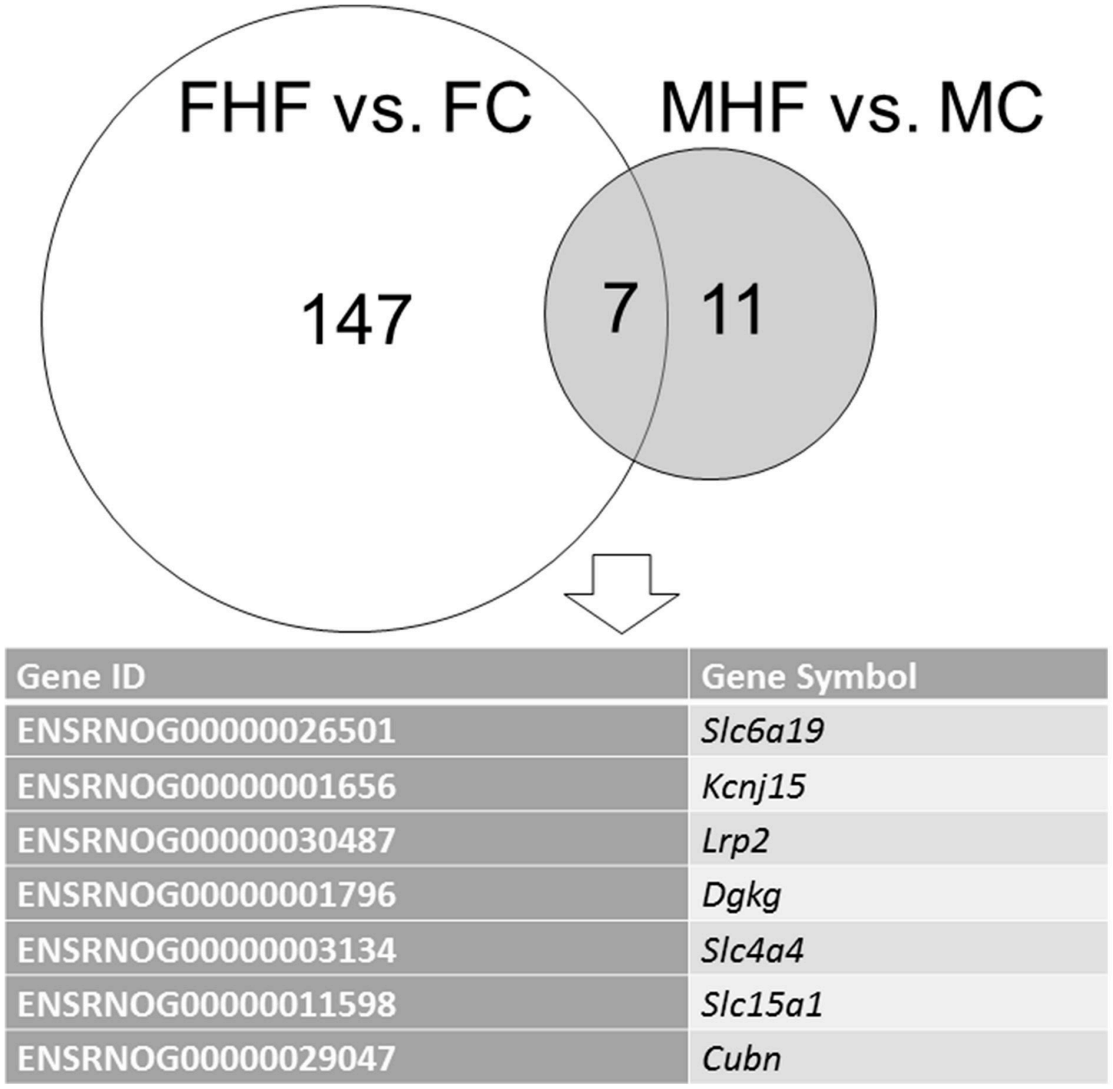
**Venn diagram depicting unique and shared (overlapping circles) sets of differential expressed genes (DEGs) between females (FHF vs. FC, white circle) and males (MHF vs. MC, gray circle)**. A total of 7 combined DEGs are listed in the lower panel.

We next analyzed differential gene expression in the kidney induced by maternal HF intake. The mappability of genes in each group compared to the rat reference genome was 78.34% for MC, 79.18% for FC, 77.32% for MHF, and 77.84% for FHF, respectively. Among the differential expressed genes (DEGs), a total of 11 genes (1 up- and 10 down-regulated genes by MHF vs. MC, Supplementary Table 2) met the selection criteria of (i) genes that changed by FPKM > 0.3 and (ii) minimum of twofold difference in normalized read counts between group. As shown in Supplementary Table 3, a total of 147 DEGs (92 up- and 55 down-regulated genes by FHF vs. FC) were noted in response to maternal HF exposure in female offspring. Among them, 7 genes, *Slc6a19, Kcnj15, Lrp2, Dgkg, Slc4a4, Slc15a1*, and *Cubn*, were shared (Figure 2). We next used DAVID v6.7 [17] to find functionally related gene groups. We observed that only one KEGG pathway related to HF-induced DEGs in the kidneys is oxidative phosphorylation (*P* = 0.015 and Benjamini value = 0.62). In addition, we found 3 HF-induced DEGs, namely *Abat, Agtr1b*, and *Hmox1*, were related to regulation of blood pressure (GO:0008271).

**Figure 3:**
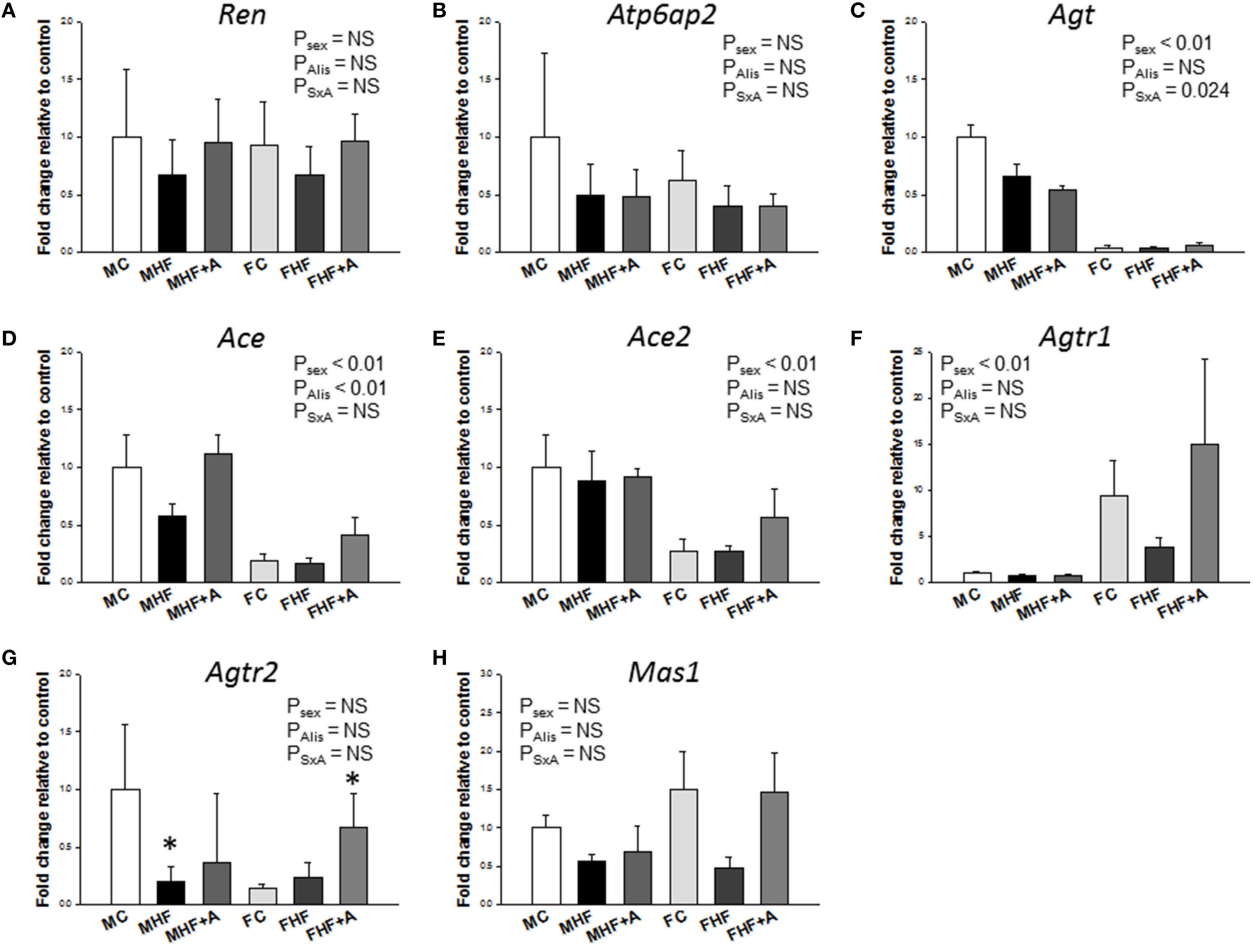
**Effects of maternal high-fructose (HF) and aliskiren administration on gene expression of renin-angiotensin system (RAS) components. (A)** *Ren*, **(B)** *Atp6ap2*, **(C)** *Agt*, **(D)** *Ace*, **(E)** *Ace2*, **(F)** *Agtr1*, **(G)** *Agtr2*, and **(H)** *Mas1* in male and female offspring kidneys at 12 weeks of age. *N* (pups/litters) = 7/4 per group; ^*^P < 0.05 vs. respective control.

Given that one HF-induced DEG with at least twofold difference between FHF vs. FC was *Agtr1b* (fold change [FC] = 4.37) (Figure 3A), and that RAS is involved in programmed hypertension, we next investigated components of RAS to elucidate underlying mechanisms related to programmed hypertension. Our NGS data demonstrated that a total of 3 genes, namely *Ren* (FC = 2.07), *Agt* (FC = 2.6), and *Mas1* (FC = 2.71) were differentially expressed between the MHF and MC group (Table 2). However, maternal HF intake altered fold changes of 4 RAS genes, *Ace* (FC = 0.31), *Ace2* (FC = 2.02), *Agtr1b* (FC = 4.37), and *Mas1* (FC = 2.77) between the FHF vs. FC group.

**Table 2:** **Fold changes in genes in renin-angiotensin system in the kidney of offspring at 1 week of age exposed to maternal high-fructose intake (HF)**.

**Gene ID**	**Gene symbol**	**MHF/MC**	**FHF/FC**	**MC/FC**	**MHF/FHF**
ENSRNOG00000002937	*Ren*	**2.07**	0.75	**0.45**	1.25
ENSRNOG00000003858	*Atp6ap2*	1.29	1.45	1.09	0.97
ENSRNOG00000018445	*Agt*	**2.60**	1.37	0.68	1.28
ENSRNOG00000007467	*Ace*	0.77	**0.31**	0.40	0.98
ENSRNOG00000031665	*Ace2*	0.96	**2.02**	1.95	0.93
ENSRNOG00000018346	*Agtr1a*	1.55	1.74	1.14	1.02
ENSRNOG00000010640	*Agtr1b*	1.49	**4.37**	**3.07**	1.04
ENSRNOG00000014971	*Mas1*	**2.71**	**2.77**	**2.14**	**2.09**

*Significant results are highlighted in bold. N = 3 per group*.

**Figure 4:**
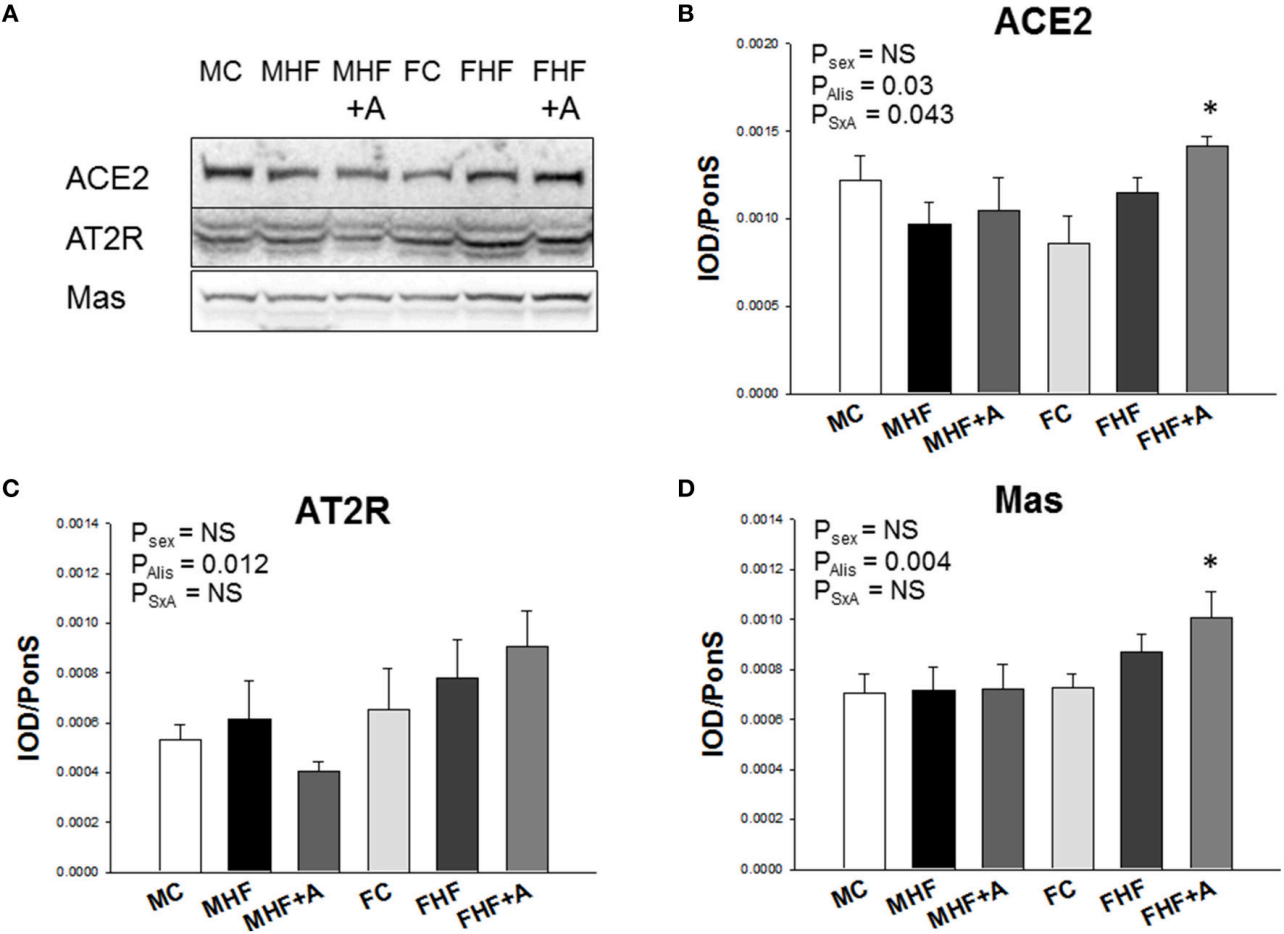
**Representative western blots (A) showed angiotensin-converting enzyme 2 (ACE2) (90 kDa), angiotensin II type 2 receptor (AT2R) (50 kDa), and angiotensin (1–7) receptor MAS (37 kDa) bands in offspring rats at 12 weeks of age**. Relative abundance of renal cortical **(B)** ACE2, **(C)** AT2R, and **(D)** MAS as quantified. *N* (pups/litters) = 7/4 per group; ^*^P < 0.05 vs. respective control.

Next, we evaluated the renal mRNA expression of RAS components at 12 weeks of age (Figure 3). In females, mRNA expression of *Agt* (*F* = 232.4; *P*_sex_ < 0.01, Figure 3C), *Ace* (*F* = 43; *P*_sex_ < 0.01, Figure 3D), and *Ace2* (*F* = 18.8; *P*_sex_ < 0.01, Figure 3E) in the kidney were lower than those in male offspring. Renal *Agtr1* mRNA expression was higher in female compared to male offspring (*F* = 95.2; *P*_sex_ < 0.01, Figure 3F). Aliskiren administration after HF intake significantly increased the renal mRNA expression of *Ace* (*F* = 9.8; *P*_Alis_ < 0.01, Figure 3D). This change was not associated with a sex-specific effect. In renal *Agtr2* mRNA expression, *post hoc* tests showed a significant difference between MC and MHF groups, and between FC and FHF+A groups. Furthermore, we analyzed the protein levels of ACE2, AT2R, and MAS in the offspring kidney (Figure 4) and found that aliskiren administration significantly increased the renal levels of ACE2 in the female offspring exposed to maternal HF intake (*F* = 4.2; *P*_SxA_ = 0.043; Figure 4B). Renal protein levels of AT2R (*F* = 7.1; *P*_Alis_ = 0.012) and MAS (*F* = 9.8; *P*_Alis_ = 0.004) were significantly higher in aliskiren treated offspring than those exposed to HF. In renal ACE2 and MAS protein level, significant differences were observed between FC and FHF+A groups.

## Discussion

This study provides insight into a novel mechanism by which aliskiren administration prevents the maternal HF-induced programmed hypertension in female adult offspring. The key findings are as follows: (1) maternal HF intake induces programmed hypertension in 12-week-old offspring of both sexes, (2) maternal HF intake induces a greater change in renal transcriptome in females than males at 1 week of age, (3) renal mRNA expression of the RAS components in differ between male and female offspring, (4) aliskiren administration during early postnatal life prevents HF-induced programmed hypertension in both sexes of adult offspring and (5) aliskiren administration increased ACE2 and MAS protein levels in female kidneys exposed to maternal HF intake.

Despite the marked hypertensive effects of HF in adults (Johnson et al., 2007; Tran et al., 2009), very few studies have considered its effects on BP when fed to pregnant dams and, subsequently, to their adult offspring (Tain et al., 2014, 2015c,d; Gray et al., 2016), especially in regard to renal programming (Tain et al., 2015d). In support of maternal HF eliciting renal programming and programmed hypertension in male offspring (Tain et al., 2014, 2015d), our study demonstrated that maternal HF induced hypertension in adult offspring of both sexes. To the best of our knowledge, the present study is the first to show that the renin inhibitor, aliskiren, during early postnatal life can prevent the development of hypertension in both sexes of adult offspring exposed to maternal HF intake. It is consistent with previous reports showing that early blockade of RAS prevents programmed hypertension in a variety of programming models (Sherman and Langley-Evans, 1998, 2000; Tain et al., 2011; Hsu et al., 2015). Interestingly, the time-dependent effect of early aliskiren on SBP is sex-specific, in that the effect after stopping treatment at 4 weeks persists up to 12 weeks in the females, but disappears between 8 and 12 weeks in the males. It has been shown that deprogramming of hypertension appears to diminish in male spontaneously hypertensive rats (SHR) while it persists in female SHR (Koeners et al., 2007), and this observation was supported by our findings demonstrating that the reduction of SBPs by aliskiren administration persisted longer in female than in male adult offspring. These findings suggest that male offspring are less amenable to reversing developmental programming of hypertension.

In line with our previous male-only study (Tain et al., 2015d), our NGS data illustrated that maternal HF intake altered renal transcriptome of both sexes at 1 week of age, with female offspring being more fructose-sensitive. Although sex differences have been observed in developmental programming of hypertension (Sandberg and Ji, 2012; Aiken and Ozanne, 2013), our study is the first to show sex differences of HF-induced changes with a focus on renal transcriptome. Very few studies have investigated sex-specifically transcriptomic change in response to maternal diet. Our NGS data demonstrated that maternal HF intake caused greater changes of renal transcriptome in females than males at 1 week of age. This finding is consistent with previous studies showing that more genes in the placenta were affected in females than in males under different maternal diets (Mao et al., 2010; Cox et al., 2013). The authors suggested that the increased female sensitivity to maternal diet might buffer the deleterious effects of such diets to protect the female fetuses, leading to a better adaptation and less impact of programming in adulthood. This concept is supported by our data showing that aliskiren administration provides better protection against hypertension programmed by maternal HF in female than male offspring with a greater reactivity of female renal transcriptome to maternal HF.

We observed 7 DEGs, *Slc6a19, Slc4a4, Slc15a1, Kcnj15, Lrp2, Dgkg*, and *Cubn*, that were shared by both sexes in response to HF exposure. Defects in three of these genes, *Slc6a19, Slc4a4*, and *Lrp2* have been linked to hypertension (Yang et al., 2012; Pinto et al., 2013; Sung et al., 2015). *Slc6a19* gene encodes an amino acid transporter B^0^AT1. *Slc4a4* gene encodes a sodium bicarbonate cotransporter (NBC) involved in the regulation of bicarbonate secretion. *Lrp2* gene encodes the multiligand receptor megalin. Further studies are needed to determine whether these genes are common genes in the development of hypertension in different programming models.

In addition, we identified three DEGs, *Abat, Hmox1*, and *Agtr1b*, related to the regulation of BP that showed sex differences in expression. *Abat* gene encodes for 4-aminobutyrate aminotransferase is responsible for catabolism of gamma-aminobutyric acid (GABA), an important inhibitory neurotransmitter. *Hmox1* encodes for heme oxygenase 1; it is critical for redox balance and is associated with hypertension. *Agtr1b* encodes for angiotensin II receptor, type 1b. It is noteworthy that maternal HF intake has a distinct sex-specific effect on the renal RAS system at 1 week of age, immediately after the completion of nephrogenesis: Maternal HF up-regulated *Ren* (FC = 2.07) and *Agt* (FC = 2.6) in male offspring, while up-regulated *Ace2* (FC = 2.02) and *Mas1* (FC = 2.77) in female offspring.

Alteration of RAS in the kidneys has been considered to be an important mechanism for the fetal programming of hypertension (Paixão and Alexander, 2013). Several RAS components can be epigenetically regulated in a maternal low protein diet model of programmed hypertension (Bogdarina et al., 2007). Despite evidence supporting sex differences in the responsiveness to Ang-(1–7) (Chappell et al., 2014), the mechanism underlying the sex-dependent effects of ACE2-Ang-(1–7)-MAS axis in HF-induced programmed hypertension have not been explored. Our results showed that aliskiren administration increased ACE2 and MAS protein levels in female kidneys exposed to maternal HF intake, which implies that the protective effect of aliskiren in hypertension programmed by maternal HF intake in females is related to the activation of ACE2-Ang-(1–7)-MAS pathway. Importantly, ACE2 appears to alter AT2R and the angiotensin (1–7) receptor MAS in a way that opposes the development of hypertension (Fraga-Silva et al., 2013). Thus, one might expect aliskiren administration during early postnatal life to upregulate AT2R and MAS, thereby affecting the RAS in a way that opposes the development of hypertension in female adult offspring. Although we observed that renal *Agt, Ace, Ace2*, and *Agtr1* expression are sex-specific, only *Ace* was significantly altered by akiskiren administration without a sex-specific effect. Consistent with a previous report showing that hepatic *Agt* mRNA levels were lower in female than in male spontaneously hypertensive rats (Chen et al., 1992), our data showed that renal *Agt* mRNA expression was lower in female than that in male SD offspring. Whether sex-specific expression of RAS components is organ-specific or species-specific awaits further evaluation. Given that *Ace2* and *Agtr2* are X-chromosome-located RAS genes (Te Riet et al., 2015), whether sex chromosomes influence their expression to protect females against HF-induced programmed hypertension deserves further clarification. Our findings in conjunction with others suggest sex-dependent renal programming within the RAS may, in part, underlie the programmed hypertension associated with HF exposure and reprogramming strategy.

One limitation in this study is that we did not analyze other organs involved in BP regulation, such as the brain and vasculature. A second limitation is that we did not examine renal transcriptome in different developmental windows. It is possible that epigenetic regulation occurs early during fetal development, and our NGS results might be a secondary phenomenon. Third, we did not conduct the MC+Aliskiren and FC+Aliskiren groups. Thus, the long-term effects of aliskiren on normal controls deserve further evaluation.

In conclusion, maternal HF induces sex-specific renal programming in the offspring. Although HF programs hypertension in the offspring of both sexes, different regulation of RAS after early aliskiren administration sex-specifically alleviates hypertension in adult offspring. It is thought that by exploration of sex differences in the susceptibility to hypertension programmed by maternal HF consumption, and of the adaptive effects that alleviate it might lead to the development of strategies to prevent programmed hypertension in both sexes.

## Author contributions

Conception and design: YT, CH, and JC. Animal treatment, collection and measurements: YT, CH, KW, and WL. Analysis and interpretation of data: YT, CH, SL, and JC. Drafting and/or revising the article critically for important intellectual content: YT and CH. Approved the final version of the manuscript: YT, CH, KW, WL, SL, and JC.

## Funding

This work was supported by grants (CMRPG8E0191 and CMRPG8F0021) from Chang Gung Memorial Hospital, Kaohsiung, Taiwan.

### Conflict of interest statement

The authors declare that the research was conducted in the absence of any commercial or financial relationships that could be construed as a potential conflict of interest.

## Abbreviations

ACE2: angiotensin converting enzyme 2
ACEI: ACE inhibitor
ARB: angiotensin receptor blocker
AT2R: angiotensin II type 2 receptor
DEG: differential expressed gene
FPKM: fragment per kilobase of exon per million mapped fragment
HF: high-fructose
KEGG: Kyoto Encyclopedia of Genes and Genomes
NGS: next-generation sequencing
RAS: renin-angiotensin system
SD: Sprague-Dawley.
