# Supplementary material for: Aliskiren Administration during Early Postnatal Life Sex-Specifically Alleviates Hypertension Programmed by Maternal High Fructose Consumption
Source: Front Physiol. 2016 Jul 12;7:299. doi: 10.3389/fphys.2016.00299 (PMC4941125; doi:10.3389/fphys.2016.00299)
Supplement: Supplementary file 1 [file Table1.DOCX]

Supplementary Material

Aliskiren therapy during early postnatal life prevents maternal high-fructose induced sex-specific programmed hypertension

Chien-Ning Hsu,1,2 Kay L.H. Wu,3 Wei-Chia Lee,4 Steve Leu,3 Julie Y.H. Chan,3 You-Lin Tain4,5*

*** Correspondence:** You-Lin Tain: tainyl@hotmail.com

# Supplementary Table 1

**Table S1 qPCR primers sequences**

| Gene | Forward | Reverse |
| --- | --- | --- |
| *Ren* | 5 aacattaccagggcaactttcact 3 | 5 acccccttcatggtgatctg 3 |
| *Atp6ap2* | 5 gaggcagtgaccctcaacat 3 | 5 ccctcctcacacaacaaggt 3 |
| *Agt* | 5 gcccaggtcgcgatgat 3 | 5 tgtacaagatgctgagtgaggcaa 3 |
| *Ace* | 5 caccggcaaggtctgctt 3 | 5 cttggcatagtttcgtgaggaa 3 |
| *Ace2* | 5 acccttcttacatcagccctactg 3 | 5 tgtccaaaacctaccccacatat 3 |
| *Agtr1* | 5 gctgggcaacgagtttgtct 3 | 5 cagtccttcagctggatcttca 3 |
| *Agtr2* | 5 caatctggctgtggctgactt 3 | 5 tgcacatcacaggtccaaaga 3 |
| *Mas1* | 5 catctctcctctcggctttgtg 3 | 5 cctcatccggaagcaaagg 3 |
| *Rn18s* | 5 gccgcggtaattccagctcca 3 | 5 cccgcccgctcccaagatc 3 |

*Ren*= Renin, *Atp6ap2*= Prorenin receptor (PRR), *Agt*= Angiotensinogen (AGT), *Ace*= Angiotensin converting enzyme (ACE), *Ace2*= Angiotensin converting enzyme (ACE2), *Agtr1* = Angiotensin II type 1 receptor (AT1R), *Agtr2* = Angiotensin II type 2 receptor (AT2R), *Mas1*= angiotensin (1-7) receptor MAS, *Rn18s* = 18S ribosomal RNA (r18S).

# Supplementary Table 2

**Table S2. Fold changes in shared genes in the kidney of MHF vs. MC at 1 week of age**

| Gene_ID | Gene Symbol | Fold changes | Log_2_ | p-Value |
| --- | --- | --- | --- | --- |
| Upregulated: 1 gene | | | | |
| ENSRNOG00000023320 | *Tspan1* | 4.059 | 2.021 | 0.0328 |
| Downregulated: 10 genes | | | | |
| ENSRNOG00000025601 | *D3ZUC6_RAT* | 0.260 | -1.943 | 0.0232 |
| ENSRNOG00000026501 | *Slc6a19* | 0.251 | -1.997 | 0.0370 |
| ENSRNOG00000001656 | *Kcnj15* | 0.221 | -2.177 | 0.0209 |
| ENSRNOG00000030487 | *Lrp2* | 0.207 | -2.270 | 0.0155 |
| ENSRNOG00000001796 | *Dgkg* | 0.185 | -2.431 | 0.0212 |
| ENSRNOG00000003134 | *Slc4a4* | 0.178 | -2.486 | 0.0039 |
| ENSRNOG00000011598 | *Slc15a1* | 0.177 | -2.501 | 0.0179 |
| ENSRNOG00000029047 | *Cubn* | 0.175 | -2.514 | 0.0311 |
| ENSRNOG00000014587 | *Mid2* | 0.112 | -3.154 | 0.0227 |
| ENSRNOG00000028008 | *Slc5a12* | 0.099 | -3.338 | 0.0024 |

# Supplementary Table 3

**Table S3. Fold changes in shared genes in the kidney of FHF vs. FC at 1 week of age**

| Gene_ID | Gene Symbol | Fold changes | Log_2_ | p-Value |
| --- | --- | --- | --- | --- |
| Upregulated: 92 genes | | | | |
| ENSRNOG00000039096 | *F1M0C9_RAT* | 9.585 | 3.261 | 0.0203 |
| ENSRNOG00000032943 | *F1LTA3_RAT* | 8.363 | 3.064 | 0.0307 |
| ENSRNOG00000017986 | *Zfp458* | 8.311 | 3.055 | 0.0246 |
| ENSRNOG00000007372 | *Casp1* | 7.786 | 2.961 | 0.0300 |
| ENSRNOG00000022218 | *D3ZAF8_RAT* | 7.649 | 2.935 | 0.0345 |
| ENSRNOG00000029602 | *F1LX69_RAT* | 7.547 | 2.916 | 0.0370 |
| ENSRNOG00000033551 | *D3ZDY7_RAT* | 7.220 | 2.852 | 0.0371 |
| ENSRNOG00000042285 | *F1LZ32_RAT* | 7.066 | 2.821 | 0.0445 |
| ENSRNOG00000028451 | *Cox7b* | 6.475 | 2.695 | 0.0137 |
| ENSRNOG00000020296 | *Usmg5* | 6.388 | 2.675 | 0.0105 |
| ENSRNOG00000009782 | *Efcab7* | 6.272 | 2.649 | 0.0153 |
| ENSRNOG00000002536 | *C1galt1c1* | 6.227 | 2.639 | 0.0071 |
| ENSRNOG00000029903 | *Spock3* | 6.116 | 2.613 | 0.0497 |
| ENSRNOG00000011817 | *Rpl22l1* | 6.108 | 2.611 | 0.0075 |
| ENSRNOG00000025768 | *Clk1* | 5.996 | 2.584 | 0.0119 |
| ENSRNOG00000002835 | *Luc7l3* | 5.911 | 2.563 | 0.0063 |
| ENSRNOG00000005858 | *Kap* | 5.828 | 2.543 | 0.0214 |
| ENSRNOG00000015410 | *Aspn* | 5.801 | 2.536 | 0.0201 |
| ENSRNOG00000026569 | *LOC100364115* | 5.784 | 2.532 | 0.0170 |
| ENSRNOG00000010408 | *LOC100361574* | 5.650 | 2.498 | 0.0067 |
| ENSRNOG00000031291 | *D3ZF90_RAT* | 5.626 | 2.492 | 0.0284 |
| ENSRNOG00000016330 | *Rbm12b* | 5.445 | 2.445 | 0.0227 |
| ENSRNOG00000001829 | *Ube2v2* | 5.442 | 2.444 | 0.0261 |
| ENSRNOG00000004419 | *D3ZVF4_RAT* | 5.363 | 2.423 | 0.0134 |
| ENSRNOG00000004754 | *D4A866_RAT* | 5.344 | 2.418 | 0.0230 |
| ENSRNOG00000009116 | *Itgb3bp* | 5.194 | 2.377 | 0.0114 |
| ENSRNOG00000010461 | *Gpx8* | 5.134 | 2.360 | 0.0117 |
| ENSRNOG00000028582 | *F1M163_RAT* | 5.117 | 2.355 | 0.0114 |
| ENSRNOG00000029792 | *Ogn* | 5.108 | 2.353 | 0.0321 |
| ENSRNOG00000026643 | *Chordc1* | 5.049 | 2.336 | 0.0150 |
| ENSRNOG00000005975 | *LOC100364549* | 5.037 | 2.333 | 0.0337 |
| ENSRNOG00000032925 | *LOC100360432* | 5.026 | 2.330 | 0.0140 |
| ENSRNOG00000017318 | *Zfp322a* | 4.999 | 2.322 | 0.0349 |
| ENSRNOG00000009841 | *E9PTD6_RAT* | 4.981 | 2.316 | 0.0199 |
| ENSRNOG00000007124 | *Krcc1* | 4.857 | 2.280 | 0.0150 |
| ENSRNOG00000008309 | *Rpa3* | 4.837 | 2.274 | 0.0165 |
| ENSRNOG00000030894 | *Slco1a6* | 4.831 | 2.272 | 0.0142 |
| ENSRNOG00000009462 | *Ccdc90b* | 4.818 | 2.268 | 0.0436 |
| ENSRNOG00000030237 | *Cox7c* | 4.813 | 2.267 | 0.0327 |
| ENSRNOG00000010921 | *Taf1d* | 4.806 | 2.265 | 0.0176 |
| ENSRNOG00000018208 | *D3ZCM1_RAT* | 4.733 | 2.243 | 0.0182 |
| ENSRNOG00000015711 | *Cetn3* | 4.728 | 2.241 | 0.0197 |
| ENSRNOG00000012512 | *NEXN_RAT* | 4.706 | 2.234 | 0.0480 |
| ENSRNOG00000014618 | *Med31* | 4.670 | 2.223 | 0.0165 |
| ENSRNOG00000015332 | *Thoc1* | 4.656 | 2.219 | 0.0168 |
| ENSRNOG00000006121 | *D3ZIB7_RAT* | 4.638 | 2.214 | 0.0259 |
| ENSRNOG00000000891 | *F1LYH2_RAT* | 4.636 | 2.213 | 0.0429 |
| ENSRNOG00000010807 | *Cox6c* | 4.628 | 2.210 | 0.0360 |
| ENSRNOG00000028328 | *Tyw3* | 4.548 | 2.185 | 0.0435 |
| ENSRNOG00000005123 | *Ttc35* | 4.413 | 2.142 | 0.0202 |
| ENSRNOG00000001317 | *Zfp68* | 4.399 | 2.137 | 0.0209 |
| ENSRNOG00000010640 | *Agtr1b* | 4.367 | 2.127 | 0.0444 |
| ENSRNOG00000011008 | *Bet1* | 4.349 | 2.121 | 0.0285 |
| ENSRNOG00000015523 | *RGD1308147* | 4.304 | 2.106 | 0.0323 |
| ENSRNOG00000024107 | *LOC690012* | 4.295 | 2.103 | 0.0484 |
| ENSRNOG00000042126 | *Lsm5* | 4.253 | 2.089 | 0.0300 |
| ENSRNOG00000006744 | *D3ZNF3_RAT* | 4.211 | 2.074 | 0.0479 |
| ENSRNOG00000002280 | *Sh3bgrl* | 4.171 | 2.060 | 0.0461 |
| ENSRNOG00000004268 | *Zfp386* | 4.161 | 2.057 | 0.0215 |
| ENSRNOG00000028585 | *Tceal8* | 4.081 | 2.029 | 0.0321 |
| ENSRNOG00000021413 | *LOC684441* | 4.081 | 2.029 | 0.0469 |
| ENSRNOG00000010891 | *Lrrcc1* | 4.074 | 2.026 | 0.0187 |
| ENSRNOG00000008504 | *Lsm8* | 4.070 | 2.025 | 0.0186 |
| ENSRNOG00000014501 | *Zfml* | 4.070 | 2.025 | 0.0335 |
| ENSRNOG00000005698 | *Ndufa5* | 4.035 | 2.012 | 0.0319 |
| ENSRNOG00000004737 | *Cd48* | 4.027 | 2.010 | 0.0343 |
| ENSRNOG00000009297 | *Chchd1* | 4.019 | 2.007 | 0.0315 |
| ENSRNOG00000006244 | *Lztfl1* | 3.948 | 1.981 | 0.0218 |
| ENSRNOG00000017469 | *Anxa1* | 3.930 | 1.975 | 0.0331 |
| ENSRNOG00000027049 | *Atp5j2* | 3.881 | 1.957 | 0.0369 |
| ENSRNOG00000002178 | *Mrps18c* | 3.866 | 1.951 | 0.0349 |
| ENSRNOG00000038960 | *RGD1309362* | 3.813 | 1.931 | 0.0374 |
| ENSRNOG00000008744 | *Cops2* | 3.813 | 1.931 | 0.0407 |
| ENSRNOG00000000120 | *Tceb1* | 3.805 | 1.928 | 0.0427 |
| ENSRNOG00000022845 | *Cep70* | 3.754 | 1.908 | 0.0490 |
| ENSRNOG00000009156 | *Tra2a* | 3.751 | 1.907 | 0.0448 |
| ENSRNOG00000030520 | *Sarnp* | 3.718 | 1.894 | 0.0453 |
| ENSRNOG00000003865 | *Tmigd1* | 3.716 | 1.894 | 0.0462 |
| ENSRNOG00000025388 | *LOC100363539* | 3.666 | 1.874 | 0.0447 |
| ENSRNOG00000032274 | *-* | 3.653 | 1.869 | 0.0292 |
| ENSRNOG00000025981 | *Ska2* | 3.621 | 1.856 | 0.0454 |
| ENSRNOG00000020315 | *Taf13* | 3.619 | 1.855 | 0.0451 |
| ENSRNOG00000031353 | *Ust5r* | 3.605 | 1.850 | 0.0423 |
| ENSRNOG00000006926 | *Atp6v0d2* | 3.571 | 1.836 | 0.0327 |
| ENSRNOG00000024372 | *Cwf19l2* | 3.566 | 1.834 | 0.0490 |
| ENSRNOG00000013002 | *Gpbp1* | 3.543 | 1.825 | 0.0380 |
| ENSRNOG00000043394 | *LOC100365201* | 3.497 | 1.806 | 0.0421 |
| ENSRNOG00000008891 | *Ift20* | 3.442 | 1.783 | 0.0377 |
| ENSRNOG00000000824 | *Dse* | 3.356 | 1.747 | 0.0399 |
| ENSRNOG00000032825 | *LOC100365668* | 3.301 | 1.723 | 0.0451 |
| ENSRNOG00000010453 | *Klhl7* | 3.184 | 1.671 | 0.0495 |
| ENSRNOG00000009888 | *Timm8b* | 3.163 | 1.661 | 0.0478 |
| Downregulated: 55 genes | | | | |
| ENSRNOG00000014530 | *Nav2* | 0.331 | -1.597 | 0.0466 |
| ENSRNOG00000009957 | *Slc25a13* | 0.327 | -1.612 | 0.0413 |
| ENSRNOG00000020440 | *Fads2* | 0.314 | -1.672 | 0.0495 |
| ENSRNOG00000006718 | *Rbm33* | 0.306 | -1.707 | 0.0391 |
| ENSRNOG00000033206 | *Entpd5* | 0.295 | -1.763 | 0.0368 |
| ENSRNOG00000030121 | *Enpep* | 0.289 | -1.789 | 0.0355 |
| ENSRNOG00000008322 | *E9PTI4_RAT* | 0.281 | -1.829 | 0.0410 |
| ENSRNOG00000004919 | *Gns* | 0.281 | -1.832 | 0.0467 |
| ENSRNOG00000001656 | *Kcnj15* | 0.279 | -1.843 | 0.0308 |
| ENSRNOG00000000555 | *Eif4ebp2* | 0.271 | -1.882 | 0.0474 |
| ENSRNOG00000014613 | *Ddah1* | 0.267 | -1.906 | 0.0250 |
| ENSRNOG00000000488 | *Hmga1* | 0.263 | -1.927 | 0.0405 |
| ENSRNOG00000023587 | *DHTK1_RAT* | 0.263 | -1.929 | 0.0232 |
| ENSRNOG00000019183 | *Alox15* | 0.259 | -1.948 | 0.0397 |
| ENSRNOG00000008988 | *Cyp2d2* | 0.259 | -1.950 | 0.0391 |
| ENSRNOG00000017762 | *Mucdhl* | 0.258 | -1.955 | 0.0448 |
| ENSRNOG00000013419 | *Agphd1* | 0.255 | -1.973 | 0.0378 |
| ENSRNOG00000000187 | *Csf2rb* | 0.255 | -1.974 | 0.0367 |
| ENSRNOG00000033932 | *-* | 0.254 | -1.977 | 0.0351 |
| ENSRNOG00000014311 | *Slc7a8* | 0.247 | -2.017 | 0.0178 |
| ENSRNOG00000001796 | *Dgkg* | 0.247 | -2.017 | 0.0224 |
| ENSRNOG00000010296 | *Slc7a7* | 0.245 | -2.030 | 0.0194 |
| ENSRNOG00000003835 | *Slc43a2* | 0.244 | -2.036 | 0.0429 |
| ENSRNOG00000000590 | *Naglt1* | 0.243 | -2.042 | 0.0186 |
| ENSRNOG00000006787 | *Dhcr24* | 0.240 | -2.061 | 0.0276 |
| ENSRNOG00000002636 | *Abat* | 0.239 | -2.066 | 0.0257 |
| ENSRNOG00000025167 | *Sema4b* | 0.237 | -2.074 | 0.0282 |
| ENSRNOG00000000245 | *Slc16a6* | 0.234 | -2.098 | 0.0466 |
| ENSRNOG00000007034 | *Hipk2* | 0.233 | -2.101 | 0.0138 |
| ENSRNOG00000030355 | *LOC100125385* | 0.232 | -2.105 | 0.0348 |
| ENSRNOG00000004882 | *Capn6* | 0.230 | -2.118 | 0.0231 |
| ENSRNOG00000010451 | *Slc34a3* | 0.221 | -2.180 | 0.0110 |
| ENSRNOG00000000168 | *Gatm* | 0.220 | -2.181 | 0.0492 |
| ENSRNOG00000015260 | *Lpar3* | 0.216 | -2.213 | 0.0456 |
| ENSRNOG00000017917 | *Pcdh24* | 0.206 | -2.281 | 0.0156 |
| ENSRNOG00000022054 | *Paqr7* | 0.205 | -2.285 | 0.0200 |
| ENSRNOG00000009448 | *D3ZD40_RAT* | 0.201 | -2.315 | 0.0136 |
| ENSRNOG00000026501 | *Slc6a19* | 0.189 | -2.403 | 0.0084 |
| ENSRNOG00000003134 | *Slc4a4* | 0.179 | -2.478 | 0.0090 |
| ENSRNOG00000015835 | *Cacna2d2* | 0.174 | -2.519 | 0.0495 |
| ENSRNOG00000026177 | *D4A3J6_RAT* | 0.171 | -2.545 | 0.0319 |
| ENSRNOG00000017438 | *Gfra1* | 0.170 | -2.559 | 0.0087 |
| ENSRNOG00000002889 | *Afp* | 0.168 | -2.574 | 0.0235 |
| ENSRNOG00000031333 | *-* | 0.161 | -2.634 | 0.0066 |
| ENSRNOG00000028274 | *D4A352_RAT* | 0.157 | -2.669 | 0.0171 |
| ENSRNOG00000030487 | *Lrp2* | 0.157 | -2.673 | 0.0093 |
| ENSRNOG00000002832 | *Slc16a2* | 0.155 | -2.685 | 0.0228 |
| ENSRNOG00000011598 | *Slc15a1* | 0.155 | -2.690 | 0.0178 |
| ENSRNOG00000014117 | *Hmox1* | 0.148 | -2.755 | 0.0037 |
| ENSRNOG00000015061 | *Igfals* | 0.143 | -2.810 | 0.0426 |
| ENSRNOG00000008312 | *Stra6* | 0.120 | -3.054 | 0.0069 |
| ENSRNOG00000029047 | *Cubn* | 0.091 | -3.465 | 0.0014 |
| ENSRNOG00000006709 | *Pzp* | 0.073 | -3.783 | 0.0160 |
| ENSRNOG00000008976 | *F1M014_RAT* | 0.064 | -3.957 | 0.0494 |
| ENSRNOG00000013062 | *Cyp24a1* | 0.046 | -4.437 | 0.0007 |
